# Combined Cyclosporin A and Hypothermia Treatment Inhibits Activation of BV-2 Microglia but Induces an Inflammatory Response in an Ischemia/Reperfusion Hippocampal Slice Culture Model

**DOI:** 10.3389/fncel.2019.00273

**Published:** 2019-06-25

**Authors:** Sylvia J. Wowro, Giang Tong, Jana Krech, Nele Rolfs, Felix Berger, Katharina R. L. Schmitt

**Affiliations:** ^1^Department of Congenital Heart Disease/Pediatric Cardiology, Universitäres Herzzentrum Berlin – Medical Heart Center of Charité and German Heart Institute Berlin, Berlin, Germany; ^2^Charité – Universitätsmedizin Berlin, Corporate Member of Freie Universität Berlin, Humboldt-Universität zu Berlin, and Berlin Institute of Health, Berlin, Germany

**Keywords:** cyclosporin A, hypothermia, oxygen-glucose deprivation/reperfusion, organotypic hippocampal slice culture, BV-2 microglia, primary neuron, inflammation, DAMPs

## Abstract

**Introduction:**

Hypothermia attenuates cerebral ischemia-induced neuronal cell death associated with neuroinflammation. The calcineurin inhibitor cyclosporin A (CsA) has been shown to be neuroprotective by minimizing activation of inflammatory pathways. Therefore, we investigated whether the combination of hypothermia and treatment with CsA has neuroprotective effects in an oxygen-glucose deprivation/reperfusion (OGD/R) injury model in neuronal and BV-2 microglia monocultures, as well as in an organotypic hippocampal slice culture (OHSC).

**Methods:**

Murine primary neurons, BV-2 microglia, and OHSC were pretreated with CsA and exposed to 1 h OGD (0.2% O_2_) followed by reperfusion at normothermia (37°C) or hypothermia (33.5°C). Cytotoxicity was measured by lactate dehydrogenase and glutamate releases. Damage-associated molecular patterns (DAMPs) high mobility group box 1 (HMGB1), heat shock protein 70 (Hsp70), and cold-inducible RNA-binding protein (CIRBP) were detected in cultured supernatant by western blot analysis. Interleukin-6 (IL-6), Interleukin-1α and -1β (IL-1α/IL1-β), tumor necrosis factor-α (TNF-α), monocyte chemotactic protein 1 (MCP1), inducible nitric oxide synthase (iNOS), glia activation factors ionized calcium-binding adapter molecule 1 (Iba1), and transforming growth factor β1 (TGF-β1) gene expressions were analyzed by RT-qPCR.

**Results:**

Exposure to OGD plus 10 μM CsA was sufficient to induce necrotic cell death and subsequent release of DAMPs in neurons but not BV-2 microglia. Moreover, OGD/R-induced secondary injury was also observed only in the neurons, which was not attenuated by cooling and no increased toxicity by CsA was observed. BV-2 microglia were not sensitive to OGD/R-induced injury but were susceptible to CsA-induced toxicity in a dose dependent manner, which was minimized by hypothermia. CsA attenuated IL-1β and Iba1 expressions in BV-2 microglia exposed to OGD/R. Hypothermia reduced IL-1β and iNOS expressions but induced TNF-α and Iba1 expressions in the microglia. However, these observations did not translate to the *ex vivo* OHCS model, as general high expressions of most cytokines investigated were observed.

**Conclusion:**

Treatment with CsA has neurotoxic effects on primary neurons exposed to OGD but could inhibit BV-2 microglia activation. However, CsA and hypothermia treatment after ischemia/reperfusion injury results in cytotoxic neuroinflammation in the complex *ex vivo* OHSC.

## Introduction

Therapeutic hypothermia (TH), also referred to as targeted temperature management (TTM), is a clinically established strategy for neuroprotection against ischemia/reperfusion injury. Several clinical studies in newborns and infants suffering from perinatal asphyxia and neonatal encephalopathy have demonstrated the efficacy of cooling to 33–34°C, resulting in lower mortality rates and improved neurological outcomes (Shankaran et al., 2012; Azzopardi et al., 2014). Additionally, cooling to a targeted temperature of 32–36°C has been established as a standard of care in patients after out-of-hospital cardiac arrest to reduce risk of death and improve neurological outcome (Hypothermia after Cardiac Arrest Study, 2002; Callaway et al., 2015; Nolan et al., 2015). Accumulating data also promotes hypothermia as a promising neuroprotective strategy in rodent models of traumatic brain injury (Liu et al., 2016b; Zhao et al., 2017) and stroke (Lee et al., 2016; Liu et al., 2018), but have yet to be translated to human trials. The recent Prophylactic Hypothermia Trial to Lessen Traumatic Brain Injury–Randomized Clinical Trial (POLAR-RCT) trial reported no benefits in neurological outcome at 6 months after applying early moderate hypothermia after severe traumatic brain injury (Cooper et al., 2018) and an acute ischemic stroke trial (Intravascular Cooling in the Treatment of Stroke) reported increased incidents of pneumonia and mortality in the hypothermia vs. normothermia treated group (Lyden et al., 2016).

Experimental investigations on the cytoprotective effects of hypothermia implicate a complex multi-modal response to protect from various ischemia/reperfusion injury mechanisms, including calcium influx, oxidative stress, mitochondrial dysfunction, apoptosis, excitotoxicity (neuronal death), and inflammation (Krech et al., 2017; Kurisu and Yenari, 2018), but the complete mechanism underlying hypothermia-induced neuroprotection remains to be elucidated. Additionally, these studies also did not investigate the effects of hypothermia on the injury-induced sterile inflammatory response.

In contrast to inflammation induced by pathogens, a sterile inflammatory response is induced by an acute condition, such as ischemia/reperfusion injury, in the absence of pathogens. Necrotic cells and other irritant particles are the stimuli for sterile inflammation by activating NLRP3 inflammasomes and inducing the release of interleukin-1 (IL-1), however, the complete mechanism is still not fully understood (Rock et al., 2010; Kono et al., 2014). Increasing evidence points to a sterile inflammatory response within the first few days after an ischemic insult that can exacerbate neuronal cell death (Ceulemans et al., 2010; Jin et al., 2013). Necrotic cell death due to an acute hypoxic-ischemic incident results in the release of DAMPs into the extracellular matrices, where they bind to pattern recognition receptors (e.g., TLRs) on local immunocompetent glial cells and initiate a neuroinflammatory response (Gulke et al., 2018).

CsA is an immunosuppressor used primarily in transplantation medicine to suppress the activation of T-lymphocytes (Borel et al., 1976). CsA binds to intracellular cyclophilin A and inhibits calcineurin, which regulates the immune response by modulating transcription factors activity (Clipstone and Crabtree, 1992). CsA has also been intensively discussed as a potential neuroprotectant as it inhibits the formation of the mitochondrial permeability transition pore by binding to cyclophilin D, thereby preventing mitochondrial dysfunction and apoptosis (Crompton, 1999; Osman et al., 2011; Fakharnia et al., 2017). Therefore, we investigated the anti-neuroinflammatory and neuroprotective effect of clinically established hypothermia in combination with CsA treatment in a murine primary neurons and BV-2 microglia monoculture model of simulated ischemia/reperfusion-induced injury. Additionally, we developed a complex murine OHSC simulated ischemia/reperfusion model where the tissue structures and cell interactions are preserved, and which is well suited for studying cell death and neuroprotective agents *ex vivo* (Li et al., 2016).

## Materials and Methods

### Cell Culture

BV-2 microglial cells are immortalized murine microglial cells (Blasi et al., 1990) with a phenotype functionally identical to native primary microglia (Henn et al., 2009), and were a kind gift from Prof. Ullrich (Zurich, Switzerland). BV-2 cells were cultured in high glucose Dulbecco’s Modified Eagle’s Medium supplemented with 1 mM pyruvate (Biochrom), 10% heat inactivated fetal bovine serum (Biochrom), and 100 U/ml penicillin/100 μg/ml streptomycin (Merck Millipore) in a 5% CO_2_ humidified atmosphere at 37°C. Cells were seeded 24 h prior to experimental start at a density of 500,000 cells in a 60 mm (21 cm^2^) dish (Sarstedt) pre-coated with 10 μg/ml PLL (Sigma-Aldrich).

### Animals and Preparation of Primary Cultures

All animal experiments were approved and performed in accordance with the guidelines of the Charité – Universitätsmedizin Berlin, Germany, and animals were housed in a conventional animal facility (FEM, Charité – Universitätsmedizin Berlin, Germany).

### Preparation of Primary Neurons

Primary neurons were prepared from embryonic day 15 (E15) C57BL/6N mice with slight modifications to the protocol previously described (Schmitt et al., 2006). Briefly, after removal of the meninges, cerebral cortices and hippocampi were digested in Hanks Balanced Salt Solution (Thermo Fisher Scientific), containing 0.2% Trypsin (Biochrom) and 100 μg/ml DNase I (Roche Diagnostics), and dissociated with a glass pipette. Cells were initially plated on 200 μg/ml PLL (Sigma-Aldrich; in 0.1 M borate buffer) pre-coated 35 mm dishes (9.2 cm^2^, TPP) in Minimum Essential Medium (MEM, Gibco) supplemented with 10% heat-inactivated horse serum (Biochrom), 6 g/L glucose (B. Braun), and 1 mg/ml Primocin (InvivoGen). The plating medium was changed to serum free Neurobasal^®^Medium (Gibco) supplemented with 1x B-27^TM^ (Gibco), 0.5 mM L-glutamine, and 1 mg/ml Primocin. After 5 days *in vitro* (DIV5) medium was changed to Neurobasal^®^ Medium supplemented with 1x B-27^TM^ minus antioxidants (Gibco). Primary neurons were cultured at 37°C in a humidified atmosphere with 5% CO_2_.

### Preparation of Organotypic Hippocampal Slice Cultures

Hippocampal slice cultures were prepared from C57BL/6N mice at postnatal day 3–5, with slight modifications to the protocol previously described (Schmitt et al., 2007). Briefly, mice were sacrificed by decapitation and the hippocampi were quickly removed and placed in ice cold MEM supplemented with 2.2 g/L sodium bicarbonate, 2 mM L-glutamine, and 8 mM Tris base (all Merck Millipore; pH 7.2). The hippocampi were transversely cut into 350 μm thick slices using a tissue chopper (McIlwain) and 6–8 slices were randomly distributed onto 30 mm membrane inserts with 0.4 μM pore size (Merck Millipore). The inserts were placed in a six-well culture dish with 1.3 ml culture medium, containing MEM, 20% heat-inactivated horse serum, 30 mM hepes (Biochrom), 2 mM L-glutamine, 2.2 g/L sodium bicarbonate, 1 μg/ml insulin (Insuman Rapid), 2.3 g/L glucose, 0.1 mg/ml Primocin, and 88 μg/ml vitamin C (Rotexmedica) at pH 7.2. Slices were cultured at 37°C in a humidified atmosphere with 5% CO_2_ for 14 days and medium was changed 1 day after preparation and every second to third day thereafter.

### Oxygen-Glucose Deprivation and Reperfusion (OGD/R)

Ischemia was simulated *in vitro* by incubation for 1 h in pre-equilibrated glucose and serum free medium at 0.2% O_2_, 5% CO_2_, and 94.8% N_2_ in a CO_2_ incubator (Binder). Control cells were maintained in glucose containing medium at Normoxia for 1 h (21% O_2_). OGD exposure in primary neurons (DIV7) and OHSC (DIV14) was conducted in glucose-free Neurobasal^®^-A Medium and in BV-2 microglia in glucose-free DMEM. After OGD, reperfusion was simulated by restoration of glucose, serum and 21% O_2_ for 24 h in all groups in appropriate complete medium.

### Time-Temperature Protocol

**FIGURE 1:**
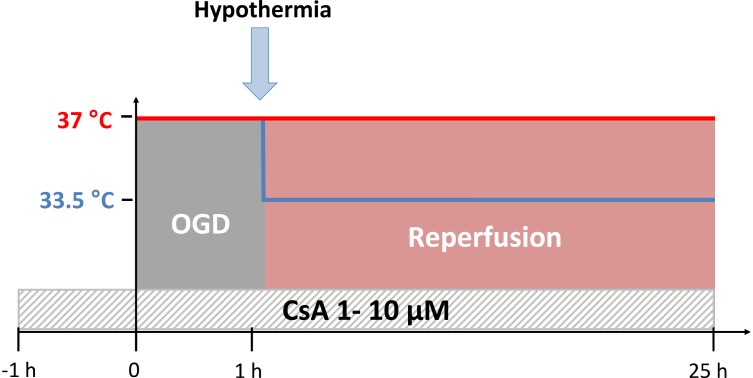
Experimental time-temperature protocol. Cyclosporin A (CsA) was applied 1 h before the simulated ischemia phase by oxygen-glucose-deprivation (OGD at 0.2% O_2_ in glucose-depleted medium) and maintained after the reperfusion phase for the duration of experiment. Exposure to OGD for 1 h was performed at 37°C, followed by reperfusion (OGD/R) for 24 h at 37°C (normothermia) or 33.5°C (hypothermia).

An experimental time-temperature protocol is illustrated in Figure 1. All cultures were exposed to OGD (0.2% O_2_) at normothermic temperature (37°C) for 1 h, followed by either normothermic (37°C) or moderate hypothermic (33.5°C) simulated reperfusion (21% O_2_) for 24 h. Control groups were maintained under normoxic and normothermic conditions (21% O_2_ and 37°C) for the duration of the experiment.

### Cyclosporin A Treatment

Cyclosporin A was purchased from Cayman (#12088). A 20 mM stock solution dissolved in ethanol (EtOH) was aliquoted and stored at −20°C. Working CsA dilutions (1 or 10 μM) in appropriate culture medium were prepared freshly and used to pre-treat cultures at 37°C and 21% O_2_ for 1 h prior to exposure to OGD and maintained throughout the duration of the experiment. Control cells were incubated with equal amount EtOH solvent.

### Assessment of Lactate Dehydrogenase (LDH) Release

Cell death was assessed by LDH released into the cultured supernatant using a colorimetric Cytotoxicity Detection Kit (Roche Diagnostics) according to the manufacturer’s instructions. Briefly, cultured supernatants were separated from cells by centrifugation and mixed with reagents (1:1) in a 96-well plate. Extinction was measured at 490 nm minus 630 nm using a microtiter plate reader (Thermo Fisher Multiskan Ascent). LDH release is expressed as a percentage of total LDH content, as determined from lysed normoxic control cells using a freeze/thaw method at −80°C.

### Assessment of Glutamate Release

Glutamate release into the cultured supernatant was measured using the Glutamat-Glo^TM^ Assay (Promega) according to the manufacturer’s instruction. Briefly, cell free supernatant was incubated with glutamate detection reagent (1:1) in a 96-well plate and luminescence signal was measured using a microplate reader (Tecan Infinite^®^M200 Pro). Glutamate concentration was extrapolated from a concentration curve and expressed in μM.

### Assessment of Propidium Iodide (PI) Staining in the OHSCs

Cell death in the OHSCs was assessed by PI staining. OHSCs were incubated with 4 μg/ml solution of PI (Sigma-Aldrich, Germany) for the last 30 min of the reperfusion phase. After staining, samples were washed twice with PBS and documented using a Keyence BZ-9000 inverted microscope.

### Extracellular Proteins Isolation and Western Blot Analysis

Supernatants were collected after 1 h of OGD or Normoxia and extracellular proteins were isolated by trichloroacetic acid precipitation. Briefly, supernatants were incubated with 20% trichloroacetic acid (VWR) for 30 min on ice, and then centrifuged at 16,000 × *g* for 20 min. The precipitated proteins were washed with ice cold aceton and dissolved in RIPA buffer. Samples were incubated with Pierce Lane Marker Reducing Sample Buffer (Thermo Scientific) at 95°C for 5 min and subjected to a 15% SDS polyacryl gel electrophoresis. Proteins were transferred onto a polyvinylidene fluoride membrane (PALL Life Sciences) overnight at 30 V using a tank blot procedure (Bio-Rad Laboratories). The membrane was blocked for 1 h at room temperature using 5% bovine albumin fraction V (Carl Roth) for Hsp70 and HMGB1 or 5% dry milk (Applied Biosystems) for CIRBP in TBS + 0.1% Tween 20. Primary antibodies against Hsp70 (1:1000, Cell Signaling Technology, Cat#4872) HMGB1 (1:1000, Chondrex, Cat#7028), and CIRBP (1:1000, Abclonal, Cat#A6080) were diluted in blocking solution and blots were incubated overnight at 4°C. HRP-conjugated secondary antibodies (anti-rabbit IgG, 1:20,000 Dianova) were incubated on the blots for 1 h at room temperature. We used Dura Super Signal West (Thermo Fisher Scientific) to visualize protein expression and ChemiDoc^TM^ Imaging Systems and Image Lab^TM^ Software (Bio-Rad) for densitometry analysis.

### RNA Isolation and Semi-Quantitative Real-Time qPCR

Total RNA from BV-2 microglia and primary neurons was isolated by acidic phenol/chloroform extraction using RNA Pure^TM^ (Peqlab) and RNA from OHSC was isolated by using the GenUP^TM^ Total RNA Kit (Biotechrabbit) followed by genomic DNA digestion using Turbo DNA-free^TM^ Kit (Ambion) according to manufacturers’ instructions, respectively. RNA concentration and purity was determined by spectrophotometric measurements at 260 nm and 280 nm using a Nanodrop 2000 (Nanodrop) and agarose gel electrophoresis. cDNA was transcribed from 1 μg total RNA using High Capacity cDNA Reverse Transcription Kit (Applied Biosystems) in a thermal cycler (PTC200, MJ Research). Expression of target genes and the endogenous control, GAPDH, was assessed by real-time qPCR using the TaqMan^®^Gene Expression Assays (summarized in Table 1) and StepOnePlus^TM^ Real-Time PCR System (Applied Biosystems) according to manufacturer’s recommendations. Reactions with no templates and RNA control were included as negative controls. Relative quantification of gene expression was normalized to the housekeeping gene GAPDH, using the 2^−ΔΔct^ method, and illustrated as fold change (Livak and Schmittgen, 2001).

**Table 1:** TaqMan^®^Gene Expression Assays.

Gene	Assay ID
GAPDH	99999915_g1
Iba1	00479862_g1
IL-1β	00434228_m1
IL-1α	00439620_m1
IL-6	00446190_m1
iNos	00440502_m1
MCP1 (Ccl2)	00441242_m1
TGF-β1	01178820_m1
TNF-α	00443260_g1

### Statistical Analysis

Experimental data from at least four independent experiments were analyzed using GraphPad Prism 7 (GraphPad Software, La Jolla, CA, United States). Values are presented as box-and-whiskers plot (box from 25th to 75th percentile and whisker min to max). Comparisons between experimental groups were made using one-way ANOVA followed by the Tukey post-test for multiple comparisons and *p* < 0.05 was considered to be significant.

## Results

Cyclosporin A is an immunosuppressant medication that has been reported to exhibit neuroprotective properties in multiple experimental models (Trumbeckaite et al., 2013). Therefore, we investigated the potential neuroprotective effect of pre-treatment with CsA (1 and 10 μM) in a simulated ischemia/reperfusion-induced injury model (1 h OGD followed by 24 h restoration of oxygen, glucose, and serum) in either a monoculture of murine primary neurons (DIV7) prepared from E15 mice, BV-2 microglia, or in an OHSC (DIV14) prepared from early postnatal (P3–5) mice that retains the cytoarchitecture and synaptic circuits of the hippocampus. Additionally, moderate hypothermia (33.5°C) was applied post-OGD to both, the *in vitro* and *ex vivo* models, to investigate any potential additive neuroprotective effects of the combined treatment.

### Cytotoxicity in Primary Neuron and Microglia Monocultures

**FIGURE 2:**
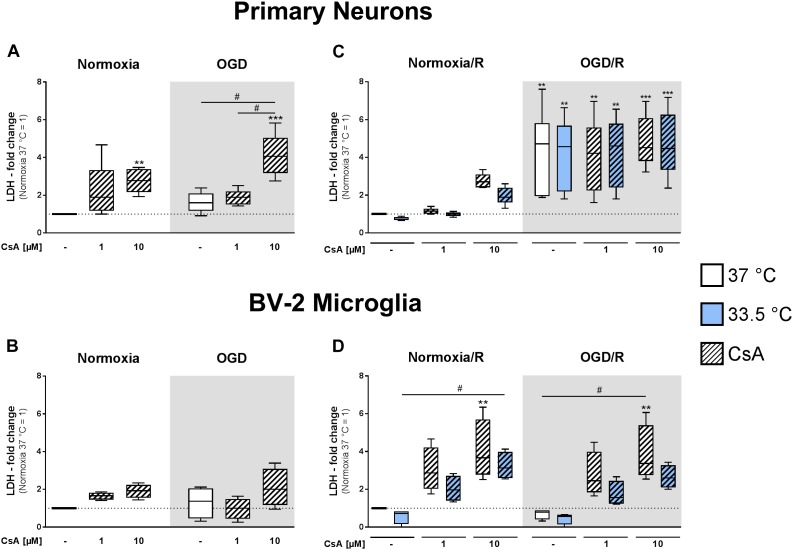
Lactate dehydrogenase releases were measured from primary neurons **(A)** and BV-2 microglia cells **(B)** in the experimental control group exposed to 1 h normoxia (21% O_2_ in glucose-containing medium) and simulated ischemia group exposed to 1 h oxygen-glucose deprivation (OGD at 0.2% O_2_ in glucose-depleted medium) at 37°C, and also after reperfusion from primary neurons **(C)** and BV-2 microglia cells **(D)** exposed to 1 h normoxia + 24 h reperfusion with complete medium (Normoxia/R) or 1 h OGD + 24 h reperfusion (OGD/R) at either 37 or 33.5°C. All CsA groups were pre-treated 1 h before experimental start with either 1 or 10 μM CsA at 37°C. Data from 4 to 6 individual experiments are presented as box-and-whiskers plot (box from 25th to 75th percentile and whisker min to max). Statistical analysis were conducted using one-way ANOVA followed by the Tukey *post hoc* test; ^∗∗^*p* < 0.01, ^∗∗∗^*p* < 0.001 compared to Normoxia at 37°C, and ^#^*p* < 0.05 for group comparison were considered significant.

We observed that initial exposure to OGD for 1 h did not significantly induce necrotic cell death compared to normoxic control in the primary neuronal and BV-2 microglial cell cultures, as measured by LDH release (Figure 2A,B). However, treatment with 10 μM CsA under both normoxic and OGD conditions resulted in measurable toxicity in the primary neurons, as observed by significantly increased LDH release compared to Normoxia control (2.8-fold and 4.1-fold increase, respectively), while 1 μM CsA was not toxic (Figure 2A). Furthermore, BV-2 microglia were resistant to CsA-induced toxicity, as no significant increases in LDH releases were measurable after exposure to 1 h OGD and treatment with 1 and 10 μM CsA (Figure 2B).

Exposure to 1 h OGD followed by 24 h reperfusion (OGD/R) resulted in a significantly higher LDH release (4.4-fold increase relative to Normoxia/R control) in the primary neurons (Figure 2C). Treatment with 1 and 10 μM CsA had no additive cytotoxic effect and necrotic cell death was not attenuated by cooling. In contrast, BV-2 microglia exposed to OGD/R showed no observable necrotic cell death, but treatment with CsA was observed to be toxic in a dose dependent manner under both Normoxia/R and OGD/R conditions, as measurable by an incremental increase in LDH release that was significant with 10 μM CsA (Normoxia/R: 4.0-fold; OGD/R: 3.8-fold) (Figure 2D). Treatment with hypothermia resulted in only slight decreases in LDH releases that did not reach significancy under all test conditions.

Interestingly, longer exposure to CsA [1 h (Figure 2B) vs. 1+24 h (Figure 2D)] induced higher LDH release in BV-2 microglia, which was not observable in the neuronal cultures. BV-2 exposed to 1 μM CsA under normoxic conditions resulted in an increase of LDH release from 1.6-fold to 3.0-fold, and 10 μM CsA resulted in an increase from 1.9-fold to 4.0-fold, respectively (Figure 2B,D).

### Cytotoxicity in Organotypic Hippocampal Slice Cultures

**FIGURE 3:**
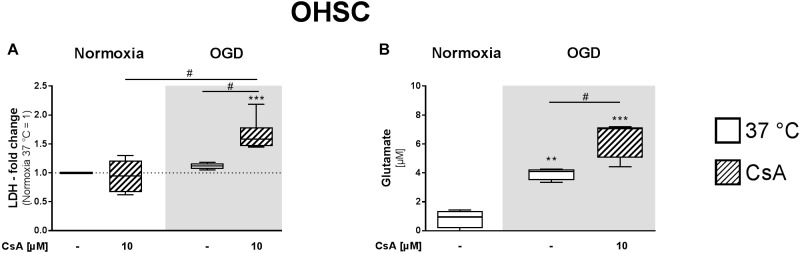
**(A)** LDH and **(B)** glutamate releases from OHSCs were assessed from the cultured supernatants of the experimental control group exposed to 1 h normoxia (21% O_2_ in glucose-containing medium) and simulated ischemia group exposed to 1 h oxygen-glucose deprivation (OGD at 0.2% O_2_ in glucose-depleted medium) at 37°C. All CsA containing groups were pre-treated 1 h before experimental start with 10 μM CsA at 37°C. Data from 4 to 5 individual experiments are presented as box-and-whiskers plot (box from 25th to 75th percentile and whisker min to max). Statistical analysis were conducted using one-way ANOVA followed by the Tukey *post hoc* test; ^∗∗^*p* < 0.01, ^∗∗∗^*p* < 0.001 compared to Normoxia at 37°C, and ^#^*p* < 0.05 for group comparison were considered significant.

Organotypic hippocampal slice culture are a suitable model for studying cell death and neuroprotective agents *ex vivo*, as the complex cellular structures and cell interactions are preserved. Therefore, we further investigated the cytotoxic effects of exposure to 1 h OGD and treatment with 10 μM CsA in OHSCs prepared from early postnatal (P3–5) mice and cultivated for 14 days (DIV14). Necrotic cell death was assessed by the release of LDH and glutamate, a neuronal excitotoxicity marker. We did not observe a significant increase in LDH release after 1 h exposure to OGD or treatment with 10 μM CsA under normoxic condition alone. However, treatment with 10 μM CsA under OGD condition resulted in an increase in LDH release that was significantly higher than for both treatments with normoxic CsA and OGD alone (Figure 3A). Interestingly, we observed significantly higher glutamate release in the OHSC exposed to 1 h OGD compared to the normoxic control (3.9 vs. 0.8 μM, respectively) (Figure 3B). Significantly higher release of glutamate was also observed in OHSC treated with 10 μM CsA under OGD condition compared to normoxic control (6.42 vs. 0.83 μM, respectively), confirming our LDH assessment indicating necrosis in the OHSCs.

**FIGURE 4:**
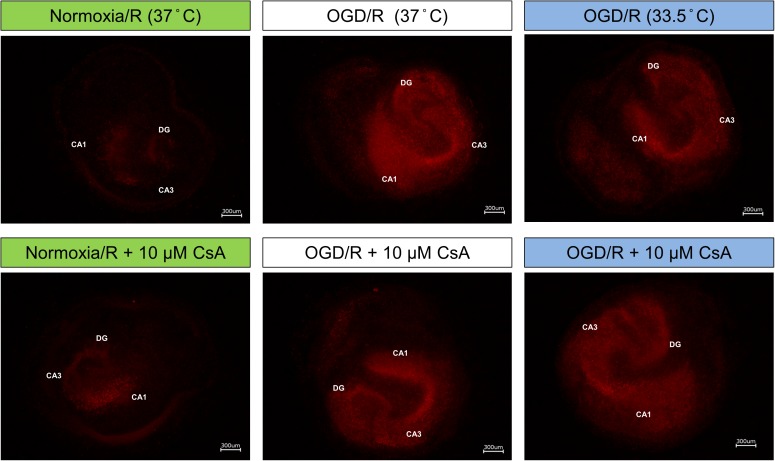
Propidium iodide (PI) staining; OHSCs in the experimental control group (Normoxia/R) were exposed to 1 h normoxia (21% O_2_ in glucose-containing medium) + 24 h reperfusion (21% O_2_ in glucose-containing medium) and simulated ischemia groups were exposed to 1 h oxygen-glucose deprivation (OGD at 0.2% O_2_ in glucose-depleted medium) + 24 h reperfusion (OGD/R at 21% O_2_ in glucose-containing medium) at either 37 or 33.5°C. All cyclosporin A (CsA) containing groups were pre-treated 1 h before experimental start with 10 μM CsA at 37°C and maintained throughout the duration of the experiment. Images are shown at 40× magnification. CA, Cornu Ammonis; DG, Dentate Gyrus.

Due to high serum concentration in the reperfusion medium, LDH and glutamate levels were not detectable after OGD/R-induced injury. Therefore, we performed PI staining to assess necrotic cell death in the OHSC after reperfusion (Figure 4). Exposure of the OHSCs to OGD/R at 37°C resulted in a marked increase in PI positive cells, as compared to Normoxia/R at 37°C. OHSCs exposed to OGD/R at 33.5°C resulted in less observable PI positive cells. OHSCs exposed to Normoxia/R +10 μM CsA at 37°C also showed a marginal increased in PI positive cells that dramatically increased when CsA was applied in combination with OGD/R at 37°C. No observable differences in PI positive cells were seen between OGD/R and OGD/R+CsA slices incubated at 37°C, nor with OGD/R+CsA slices incubated at 33.5°C.

### Release of DAMPs

**FIGURE 5:**
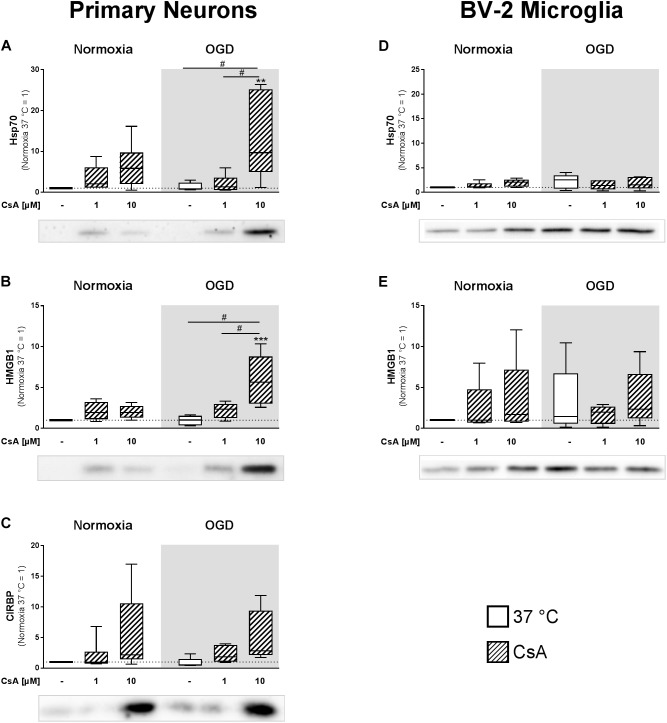
Western Blot analysis was used to assess extracellular DAMPs, **(A,D)** heat shock protein 70 (Hsp70), **(B,E)** high mobility group box 1 (HMGB1), and **(C)** cold-inducible RNA-binding protein (CIRBP) released into the cultured supernatant from primary neurons and BV-2 microglial cells. The experimental control group was exposed to 1 h normoxia (21% O_2_ in glucose-containing medium) and simulated ischemia group was exposed to 1 h oxygen-glucose deprivation (OGD at 0.2% O_2_ in glucose-depleted medium) at 37°C. Quantitative densitometric analysis from 5 to 6 individual experiments is presented as box-and-whiskers plot (box from 25th to 75th percentile and whisker min to max), along with the representative immunoblots. Statistical analysis were conducted using one-way ANOVA followed by the Tukey *post hoc* test; ^∗∗^*p* < 0.01, ^∗∗∗^*p* < 0.001 compared to normoxia at 37°C, and ^#^*p* < 0.05 for group comparison were considered significant.

Soluble inflammatory mediators are released from the necrotic brain tissue. Therefore, we isolated extracellular proteins from cell-cultured supernatants to investigate the release of inflammation inducing DAMPs from primary neurons and BV-2 microglia exposed to 1 h OGD. Correlating with the LDH measurements, western blot analysis showed significantly higher releases of HMGB1 and HSP70 in primary neurons treated with 10 μM CsA under OGD conditions, in comparison to normoxic control, as well as to all other OGD treated groups (Figure 5A,B). Additionally, we observed a trend toward a higher release of CIRBP in neurons treated with 10 μM CsA under OGD conditions (Figure 5C). Also in correlation to LDH release, no significant increases in extracellular HMGB1 and HSP70 were observed in non-necrotic BV-2 microglia exposed to OGD and treated with 10 μM CsA (Figure 5D,E). Interestingly, we observed elevated but non-significant DAMPs releases in all OGD damaged BV-2 cells in comparison to Normoxia control, which was not seen in the primary neurons. Extracellular CIRBP was not detectable in the BV-2 microglia cultured supernatant. Furthermore, none of the investigated DAMPs were detectable in the cultured supernatant of the OHSCs.

### Cytokine and Chemokine Expression in BV-2 Microglia, Primary Neurons, and OHSC

Based on our observation of increased DAMPs release from necrotic primary neurons, we next investigated the inflammatory response in the immune competent BV-2 microglia, in primary neurons, and OHSC after exposure to OGD/R and treatment with CsA where necrosis was detectable in neurons and OHSC. Additionally, we also investigated the effect of cooling (33.5°C) applied during the reperfusion phase on inflammatory cytokines and chemokines gene expressions.

### Cytokine Expressions in BV-2 Microglia and Primary Neurons

**FIGURE 6:**
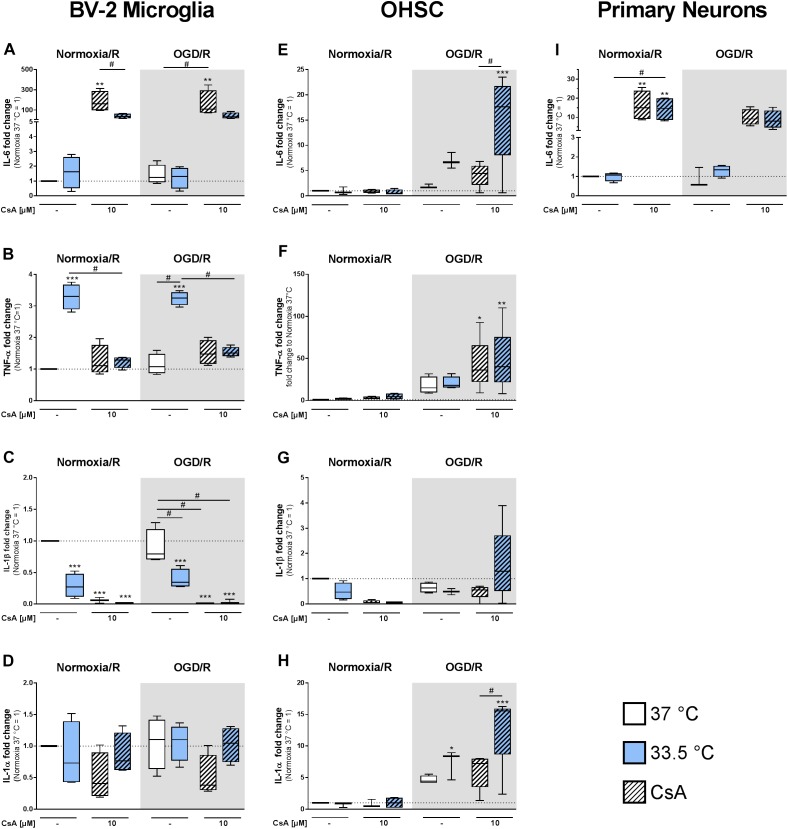
RT-qPCR was used to assess gene expressions in BV-2 microglia, OHSCs, and primary neurons for inflammatory **(A,E,I)** IL-6, **(B,F)** TNF-α, **(C,G)** IL-1β, and **(D,H)** IL-1α after exposure to Normoxia/R (1 h normoxia + 24 h reperfusion at 21% O_2_ in glucose-containing medium) or OGD/R (1 h OGD + 24 h reperfusion at 0.2% O_2_ in glucose-depleted medium) at either 37 or 33.5°C. All CsA containing groups were pre-treated with 10 μM CsA at 37°C. Data from 4 to 5 individual experiments are presented as box-and-whiskers plot (box from 25th to 75th percentile and whisker min to max). Statistical analysis were conducted using one-way ANOVA followed by the Tukey *post hoc* test; ^∗^*p* < 0.01, ^∗∗^*p* < 0.01, ^∗∗∗^*p* < 0.001 compared to Normoxia/R at 37°C, and ^#^*p* < 0.05 for group comparison were considered significant.

We observed a significant increase in IL-6 expression in the BV-2 microglia treated with 10 μM CsA under both Normoxia/R and OGD/R conditions, which correlated with the observed necrotic cell death (Figure 6A). Cooling significantly reduced IL-6 expression in CsA treated Normoxia/R control and in weaker extend in the OGD/R injury group. Similar increased IL-6 expression was also observed in the primary neurons treated with 10 μM CsA (Figure 6I). Interestingly, TNF-α expression was significantly induced by cooling under both Normoxia/R and OGD/R conditions in BV-2 microglia, which was attenuated by treatment with CsA (Figure 6B). Additionally, cooling and CsA alone and in combination significantly inhibited IL-1β expression under both Normoxia/R and OGD/R conditions (Figure 6C), but had no significant effect on IL-1α expression in BV-2 microglia (Figure 6D). TNF-α, IL-1β, and IL-1α expressions were under detection limit in the primary neurons.

### Cytokine Expressions in the OHSC Model

We next investigated if exposure to OGD/R injury and treatment with CsA had a similar cytokine expression regulation in the OHSC model as observed in the BV-2 microglia. In general, inflammatory cytokines expressions in the OHSC model did not reflect what we observed in the BV-2 microglia monoculture. Interestingly, the combined treatment of 10 μM CsA, OGD/R, and cooling resulted in the greatest general increase in cytokines expressions. Under these combined conditions, IL-6 expression was significantly higher than Normoxia/R control and OGD/R+CsA, indicating a profound effect of cooling on IL-6 expression (Figure 6E). TNF-α expression was significantly higher than Normoxia/R control in the OGD/R+CsA treated group with no observable effect of cooling (Figure 6F). IL-1β expression was not suppressed as observed in the BV-2 microglia (Figure 6G), and IL-1α expression appears to be more induced by cooling under both OGD/R and OGD/R+CsA treatments compared to 37°C Normoxia/R group (Figure 6H).

### Chemokine and Growth Factor Expressions in BV-2 Microglia and Primary Neurons

**FIGURE 7:**
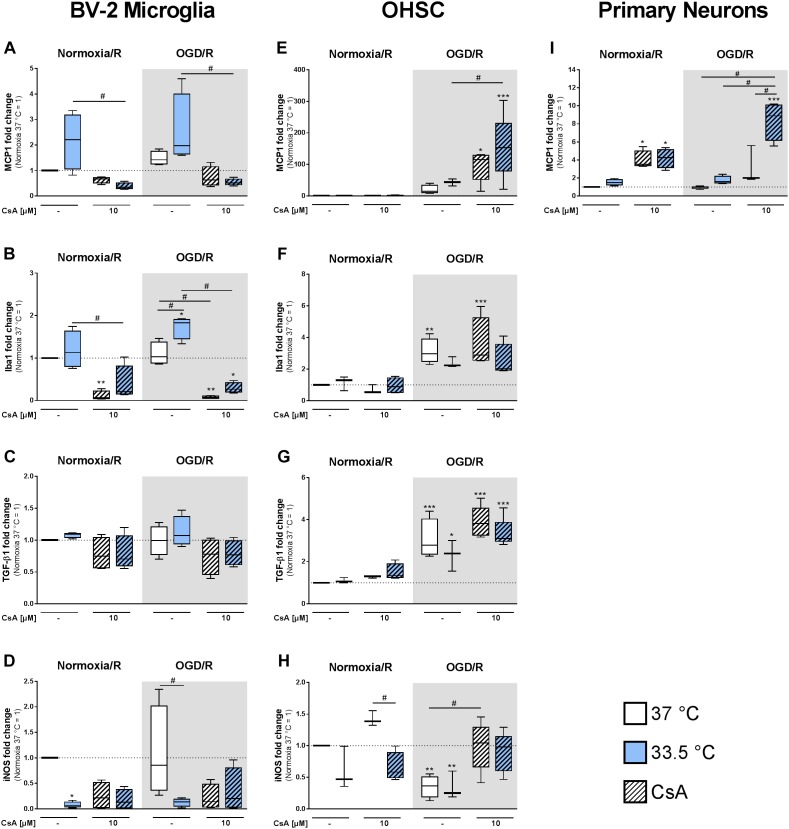
RT-qPCR was used to assess gene expressions in the BV-2 microglia, OHSCs, and primary neurons for **(A,E,I)** MCP1, **(B,F)** Iba1, **(C,G)** TGF-β1, and **(D,H)** iNOS after exposure to Normoxia/R (1 h normoxia + 24 h reperfusion at 21% O_2_ in glucose-containing medium) or OGD/R (1 h OGD + 24 h reperfusion at 0.2% O_2_ in glucose-depleted medium) at either 37 or 33.5°C. All CsA containing groups were pre-treated with 10 μM CsA at 37°C. Data from 4 to 5 individual experiments are presented as box-and-whiskers plot (box from 25th to 75th percentile and whisker min to max). Statistical analysis were conducted using one-way ANOVA followed by the Tukey *post hoc* test; ^∗^*p* < 0.01, ^∗∗^*p* < 0.01, ^∗∗∗^*p* < 0.001 compared to Normoxia/R at 37°C, and ^#^*p* < 0.05 for group comparison were considered significant.

Furthermore, we investigated the expression of targets regulating migration and activation of microglia and monocytes. In BV-2 microglia we observed a trend toward hypothermia-induced MCP1 expression, whereas treatment with CsA rather reduced MCP1 expression in comparison to Normoxia/R control (Figure 7A). In contrast to BV-2 microglia, we found an increased MCP1 expression in primary neurons treated with 10 μM CsA, which was even higher at 33.5°C in the OGD/R injured group (Figure 7I).

Activation of microglia is associated with increased Iba1 expression (Hovens et al., 2014). Similar to MCP1, we found Iba1 expression significantly induced in the cooled OGD/R injured group, while treatment with CsA significantly decreased Iba1 expression (Figure 7B). The expression of growth factor TGF-β1 was unchanged in all BV-2 microglia groups (Figure 7C). Additionally, we found iNOS expression to be significantly suppressed by cooling under Normoxia/R, and to a less extend by OGD/R at 33.5°C and by CsA treatment (Figure 7D). Iba1, TGF-β1, and iNOS expressions were under detection limit in the primary neurons.

### Chemokine and Growth Factor Expressions in OHSC

In the OHSC model we observed a strong increase in chemokine MCP1 expression in OGD/R and OGD/R+CsA compared to Normoxia/R control (Figure 7E). A similar response was also found in primary neurons (Figure 7I). Iba1 gene expression was induced in OGD/R injury alone and in combination with CsA (Figure 7F). Comparable to Iba1, the expression of growth factor TGF-β1 was increasingly expressed in OGD/R injured slices, while cooling and CsA treatment had no additional effect (Figure 7G). Interestingly, iNOS expression was significantly decreased by OGD/R-induced injury and increased to Normoxia/R level by OGD/R+CsA (Figure 7H).

## Discussion

Neuroinflammation plays a central role in the pathogenesis of ischemic brain injury (Bhalala et al., 2014; Hagberg et al., 2015). Therefore, we evaluated the anti-inflammatory and neuroprotective effect of clinically discussed hypothermia in combination with CsA immunosuppressant treatment on OGD/R injured brain cells. In our study treatment with hypothermia and CsA caused an alteration in the inflammatory pathways. While pro-inflammatory IL-1β and iNOS expressions are effectually suppressed in BV-2 microglia and OHSC, expression of TNF-α, IL-1α, MCP1, Iba1, and TGF-β1 are increased in OGD/R injured OHSC. We found that primary neurons undergo cell death by OGD/R-induced injury and by CsA treatment, suggesting a complex activation of inflammatory pathways through the involvement of a secondary damage mechanism in complex slice culture. Our findings demonstrate that CsA, a highly specific inhibitor of calcineurin, affects the survival of primary neurons and modulate the inflammatory response in microglial cultures, as well as in the OHSC.

### Cytotoxicity-Induced DAMPs Release

Primary neurons pre-treated with 10 μM CsA followed by 1 h exposure to OGD undergo necrotic cell death, as indicated by increased LDH release. Additionally, increased amounts of DAMPs, namely Hsp70, HMGB1, and CIRBP were detected in the cultured supernatants. Hsp70 and HMGB1 are classical DAMPs and have been extensively investigated (Asea et al., 2000; Muhammad et al., 2008), whereas the inflammation-inducing DAMP property of extracellular CIRBP is a recent observation that has been shown to perpetuate the inflammatory response in hemorrhagic shock and sepsis patients (Qiang et al., 2013). Although both, intracellular Hsp70 and CIRBP, have been shown to have desirable anti-apoptotic properties, their presence in the extracellular matrix has been shown to initiate inflammatory responses (Liao et al., 2017; Kim et al., 2018). Their affinity to bind to surface receptors, including RAGE, toll-like receptor 2 (TLR2), and 4 (TLR4) on adjacent cells leads to the activation of the nuclear factor “kappa-light-chain-enhancer” of activated B-cells (NF-κB) pathway and transcription of inflammatory cytokines, such as TNF-α, IL-1β, and IL-6 (Johnson and Fleshner, 2006; Andersson and Tracey, 2011; Zhou et al., 2014). In comparison to neurons, BV-2 microglia were more vulnerable to OGD-induced release of HMGB1 and Hsp70, which did not reach significance, but were less vulnerable to OGD+CsA-induced cytotoxicity, which resulted in no additional DAMPs release.

Organotypic hippocampal slice culture exposed to 1 h of OGD had a significant increased release of glutamate, but no increase in LDH release was measureable. Under non-pathological conditions glutamate in the extracellular space is cleared by astrocytes via glutamate transporters, but under ischemic conditions the uptake is reversed due to ion gradients changes, resulting in the release of glutamate to the extracellular space. High concentration of glutamate can cause extensive neuronal injury and subsequently trigger cell death (Rossi et al., 2000; Nishizawa, 2001; Zhang et al., 2019). The addition of CsA during the OGD phase resulted in an increase in LDH release and an additional increase of glutamate in the cultured supernatant, presumably by necrotic cells, as we also observed CsA-induced cell death in both microglial and neuronal mono-cultures. Our findings are in line with an *in vitro* study of primary mixed neurons-astrocytes culture showing CsA-induced cytotoxicity only in the neurons, while the astrocytes were unharmed (Kaminska et al., 2001).

Reperfusion-induced injury occurs when restoration of energy and oxygen after an OGD phase is achieved, causing alterations in multiple pathways affecting inflammation, redox-system, and many other signaling pathways (Lopez-Neblina et al., 2005; Mizuma and Yenari, 2017). In line with previous findings describing a higher resistance of microglia than neurons to OGD/R-induced injury, we also observed a marked increase in necrosis in primary neurons but absent in BV-2 microglia after exposure to OGD/R (Goldberg and Choi, 1993; Lim et al., 2006; Li et al., 2007). Surprisingly, neither treatment with moderate hypothermia for 24 h, nor CsA protected the neurons from OGD/R-induced cell death. Conversely, treatment with CsA resulted in BV-2 microglia cell death in a concentration dependent manner, which could be partially attenuated by cooling. In the *ex vivo* OHSCs, we observed a dramatic increase in PI positive cells after exposure to OGD/R at 37°C, which was decreased by cooling to 33.5°C. Similar exposure to OGD/R at 37°C also resulted in significant cell death in the primary neuronal culture. However, treatment with CsA during OGD/R had no observable protective effect in the slice cultures independent of temperature, as similar intensities in PI staining were also observed in the slices exposed to OGD/R alone. This is in contrast to Liu et al. (2016a), who reported a protective effect of CsA on the mitochondria which was augmented by hypothermia in an *in vivo* rat cardiac arrest model. Unfortunately, quantification of DAMPs release in the reperfusion phase was not possible by western blot analysis due to the high serum concentration in the cultured medium, and warrants further investigation.

### Hypothermia

To date, it is widely accepted that multiple pathways and mechanisms are involved in the protective effect of hypothermia (Kurisu and Yenari, 2018). Preclinical studies *in vitro* and *in vivo* suggest that anti-inflammatory mechanisms induced by hypothermia play an important role (Lee et al., 2016). Exposure of BV-2 microglia to hypothermia for 24 h had variable effects on known pro-inflammatory mediators, resulting in increased TNF-α and MCP1 expressions and decreased IL-1β and iNOS expressions under both Normoxia/R and OGD/R conditions. While increased iNOS and IL-1β levels are generally associated with neurotoxic effects, TNF-α also exhibits neuroprotective properties (Kawabori and Yenari, 2015). Lambertsen et al. (2009) showed in an *in vivo* cerebral ischemia model that microglia derived TNF-α enhances neuronal survival. Other *ex vivo* OHSC studies showed that deep hypothermia induces TNF-α secretion, resulting in neurite outgrowths (Schmitt et al., 2010). Nevertheless, neurotoxic effects by high levels of TNF-α in the ischemic brain injury are also well documented (Sriram and O’Callaghan, 2007). Also the expression of the chemokines MCP1 leads to recruitment and migration of leukocyte from the periphery to the site of injury. MCP1 is upregulated in models of cerebral ischemia and inhibition of MCP1 can decrease brain injury (Chen et al., 2003).

When the same OGD/R injury protocol was applied to the OHSC model *ex vivo*, neither significant induction of TNF-α and MCP1 expressions, nor a significant inhibition of IL-1β by cooling was observed. The heterogeneity of cells in the OHSC, with 5–15% of microglial cells in the hippocampus (Lawson et al., 1990) may explain the variability.

### Cyclosporin A

Cyclosporin A is a cyclophilin binding substance whose primary immunosuppressive function is to inhibit calcineurin, a Ca^2+^/calmodulin dependent protein phosphatase. Calcineurin is ubiquitously expressed in most tissues, but at particularly high concentrations in the brain. In glia cells calcineurin plays a global role in neuroinflammation, as it interacts and modulates multiple transcription factors, including NFAT, NF-κB, and AP1, which are associated with cytokines expression (Furman and Norris, 2014).

In the BV-2 microglia, cooling suppressed IL-1β and iNOS expressions, but also induced TNF-α and MCP1 expressions, which was attenuated by CsA treatment. Increased pro-inflammatory iNOS expression has been observed to result in the production of high amounts of NO, which attributes to brain cytotoxicity and promotes ischemic cell death (Amantea et al., 2009; Terpolilli et al., 2012). Zawadzka et al. (2012) observed that CsA inhibits iNOS expression in an *in vitro* model of lipopolysaccharide stimulated microglia, by interfering with the MAPK and NF-κB signaling pathways. Our findings indicate that NF-κB driven cytokines expressions are also inhibited by CsA treatment. This is not surprising as calcineurin has been shown to be indirectly involved in the activation of NF-κB by degrading IκBα, as well as NFATs regulated cytokine gene expressions by coupling with AP1 or NF-κB, amongst others (Macian et al., 2001; Sama et al., 2008; Palkowitsch et al., 2011). Multiple isoforms of NFAT that are calcineurin activated transcription factors have been shown to be expressed by microglia and can be specifically inhibited by the NFAT inhibitor VIVIT, resulting in decreased secretions of TNF-α and MCP1 (Nagamoto-Combs and Combs, 2010; Rojanathammanee et al., 2015). Additionally, microglia activation marker, Iba1, expression was also inhibited by CsA. In contrast to reduced MCP1 expression observed in the BV-2 microglia, CsA induces expression of MCP1 in the primary neuronal cultures under both Normoxia/R and OGD/R conditions. This is not surprising, as neurons have been observed to be capable of expressing chemokines, including MCP1, during the early phase after ischemia in an *in vivo* model of focal cerebral ischemia (Che et al., 2001).

Interestingly, treatment with CsA upregulated IL-6 expression, but had no measurable effect on TGF-β1 and IL-1α expressions in the BV-2 microglia. IL-6 was also the only cytokine we observed to be expressed under OGD/R+CsA treatment in primary neurons. IL-6 can be secreted by both immune (microglia) and non-immune (neurons) cells and function as a neurotrophic factor and inhibitor of neuronal death (Loddick et al., 1998). At the same time IL-6 is involved in the pathological progression of several inflammatory diseases (Rothaug et al., 2016).

Altogether, CsA exhibits an immunosuppressive effect in a BV-2 microglia model, but it also had a toxic effect at the same concentration in a murine dissociated neuronal culture. The response to OGD/R-induced injury in combination with CsA and hypothermia treatment was also investigated in a more complex *ex vivo* OHSC model, which shows a different pattern of response to simulated ischemia than the individual primary neuronal cultures and BV-2 microglia. We did not observe significant increased induction of the inflammatory pathways investigated in the OGD/R-induced injured slices, but surprisingly increases in TNF-α, IL-1α, IL-6, and MCP1 expressions were observed when 10 μM CsA was additionally introduced, which was not observed in the Normoxia/R control treated with CsA nor in the BV-2 microglia under OGD/R+CsA conditions. As astrocytes are the most abundant glial cell type in the brain capable of participating in the immune response (Becerra-Calixto and Cardona-Gomez, 2017), the observed increase in inflammatory response in our *ex vivo* OHSC model could be considered to be astrocytes driven. Increased MCP1 expression has been observed in hippocampal astrocytes after *in vivo* transient global ischemia (Sakurai-Yamashita et al., 2006), which can trigger the adaptive immunity response in the inflamed CNS (Farina et al., 2007). Additionally, *in vitro* studies with primary astrocytes showed longer exposure to OGD (24 h) resulted in increased TNF-α and IL-1β secretions, which was attenuated by CsA treatment (Gabryel et al., 2004). Moreover, *in vivo* studies of transient middle cerebral artery occlusion in a rat model found decreased TNF-α secretion after treatment with CsA containing nanoliposomes compared to non-treated animals (Partoazar et al., 2017). This is in contrast to our findings in the OHSC treated with OGD/R+CsA, indicating that the putative protective effect of CsA may be dominated by or overlaid with other pro-inflammatory mediators.

Other studies showed DAMPs released from necrotic neurons in hippocampal slices or treated with exogenous HMGB1 lead to induced expression of inflammatory cytokines TNF-α and IL-1β via TLR4 activation in microglia cells (Zou and Crews, 2014). As we found LDH and glutamate secretions are increased in OGD+CsA-induced damaged OHSC, it is likely that various DAMPs are released from necrotic neurons, thereby activating neighboring microglia and astrocytes to drive the secondary inflammatory process after OGD/R-induced injury. While CsA mediated suppression of microglia activation, it is not sufficient to reduce the overall inflammatory response in the complex OHSC. To our knowledge, the effect of CsA on the inflammatory response in OGD/R-induced OHSC injury has not been investigated, as most research studies have investigated the amelioration of cell death by CsA treatment, and protection of the mitochondria from OGD/R-induced opening of the mitochondrial permeability transition pores (Kawakami, 2013; Trumbeckaite et al., 2013; Yu et al., 2013).

An inflammatory response in the brain can be both beneficial and harmful, depending on the type and amount of cytokines expressed. An increase in Iba1 expression is associated with microglia activation (Hovens et al., 2014). TGF-β1 is mainly expressed by these activated microglial cells and is associated with a reduction of neuronal cell death and decreased infarct size after cerebral ischemia (Meyers and Kessler, 2017). We observed both Iba1 and TGF-β1 expressions to be increased in OGD/R injured slices, whereas other cytokines were not significantly altered. Our findings indicate a beneficial activation of inflammatory processes after exposure to OGD/R in the OHSC model, but it becomes highly neuroinflammatory when CsA is additionally applied.

We acknowledge several limitations of our study. First, we only assessed cytokine and chemokine mRNA expressions and not the secreted form. Therefore, any conclusion concerning inflammation should be approached with caution, as intracellular and secreted levels may differ. Second, quantifiable necrosis in the hippocampal slice cultures as well as any subsequent DAMPs release after reperfusion was not possible due to high serum concentrations in the cultured media. Therefore, further studies are needed to investigate the impact of DAMPs associated sterile inflammation in CNS cells. Finally, our protocol focused on ischemia sensitive neurons and immunocompetent microglia. As astrocytes constitute a large population of cells in the brain and have an important role in neuroprotection, further studies investigating the effects of CsA and cooling on OGD/R-induced injury in astrocytes are warranted.

## Conclusion

We found CsA treatment to be effective in suppressing inflammation in a pure microglia culture after OGD/R-induced injury, but causes necrotic cell death in primary neurons. In the complex *ex vivo* slice culture CsA treatment lead to an exacerbated immune response, which was not diminished by hypothermia but instead potentiated an additive effect leading to an increase in neuroinflammation. Based on our findings, the combination of cooling and CsA treatment can hereby not be considered as neuroprotective. In contrast to other studies describing the neuroprotective effects of CsA and hypothermia, we observed the induction of neuroinflammation to the combined treatment in a complex OHSC model.

## Data Availability

The raw data supporting the conclusions of this manuscript will be made available by the authors, without undue reservation, to any qualified researcher.

## Ethics Statement

This study was carried out in accordance with the guidelines of the Charité – Universitätsmedizin Berlin, Germany and the national ethic principles (registration no. T0044/08).

## Author Contributions

SW, GT, and KS designed the experiments. SW conducted the experiments and analyzed the data. SW, GT, KS, NR, JK, and FB participated in the discussion of the results. SW prepared the manuscript. GT, JK, NR, and KS reviewed the manuscript. All authors read and approved the final manuscript.

## Conflict of Interest Statement

The authors declare that the research was conducted in the absence of any commercial or financial relationships that could be construed as a potential conflict of interest.

## Abbreviations

AP1: activator protein 1
CIRBP: cold-inducible RNA-binding protein
CNS: central nervous system
CsA: cyclosporin A
DAMP: damage-associated molecular pattern
DIV: days *in vitro*
GAPDH: glyceraldehyde 3-phosphate dehydrogenase
HMGB1: high mobility group box 1
Hsp70: heat shock protein 70
iNOS: inducible nitric oxide synthase
Iba1: ionized calcium-binding adapter molecule 1
IL-6: interleukin-6
IL-1α: interleukin-1α
IL-1β: interleukin-1β
LDH: lactate dehydrogenase
MAPK: mitogen-activated protein kinase
MCP1: monocyte chemotactic protein 1
NFAT: nuclear factor of activated T-cells
OGD: oxygen-glucose-deprivation
OGD/R: OGD and reperfusion
OHSC: organotypic hippocampal slice culture
PI: propidium iodide
PLL: poly-L-lysin
RAGE: receptor for advanced glycation endproducts
RT-qPCR: reverse transcription quantitative polymerase chain reaction
TGF-β1: transforming growth factor-β1
TH: therapeutic hypothermia
TLR: toll-like receptor
TNF-α: tumor necrosis factor-α
TTM: targeted temperature management.
